# Query Mix-Max Method for FDR Estimation Supported by Entrapment Queries

**DOI:** 10.1021/acs.jproteome.4c00744

**Published:** 2025-02-05

**Authors:** Dominik Madej, Henry Lam

**Affiliations:** Department of Chemical and Biological Engineering, The Hong Kong University of Science and Technology, Hong Kong, China

**Keywords:** entrapment query, entrapment database, false discovery rate, peptide identification, shotgun proteomics

## Abstract

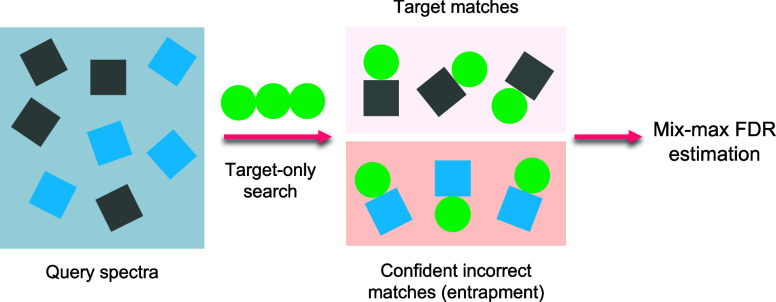

Estimating the false discovery rate (FDR) is one of the key steps in ensuring appropriate error control in the analysis of shotgun proteomics data. Traditional estimation methods typically rely on decoy sequence databases or spectral libraries, which may not always provide satisfactory results due to limitations of decoy construction methods. This study introduces the query mix-max (QMM) method, a decoy-free alternative for FDR estimation in proteomics. The QMM framework builds upon the existing mix-max procedure but replaces decoy matches with entrapment queries to estimate the number of false positive discoveries. Through simulations and real data set analyses, the QMM method was demonstrated to provide reasonably accurate FDR estimation across various scenarios, particularly when smaller sample-to-entrapment spectra ratios were achieved. The QMM method tends to be conservatively biased, particularly at higher FDR values, which can ensure stringent FDR control. While flexible, the protocol’s effectiveness may vary depending on the evolutionary distance between the sample and entrapment organisms. It also requires a sufficient number of entrapment queries to provide stable FDR estimates, especially for low FDR values. Despite these limitations, the QMM method is a promising alternative as one of the first query-based FDR estimation approaches in shotgun proteomics.

## Introduction

In shotgun proteomics, the identification and quantification of proteins found in complex biological samples rely on accurately identifying peptides produced by those proteins upon enzymatic digestion.^1^ To ensure that the set of peptides marked as correctly identified does not contain too many false positives, an appropriate error control must be executed, typically in the form of controlling the false discovery rate (FDR)—the expected proportion of false positives in the set of accepted discoveries.^2^ Accurate FDR estimation is necessary, as it directly impacts the quality of the results used to draw final biological conclusions.

Currently, the FDR in shotgun proteomics data analysis is typically estimated using either decoy-based or decoy-free approaches. Perhaps the most popular of all is target-decoy competition (TDC), which involves generating a set of decoy candidates (sequences or spectra) that do not occur in nature and searching them together with the target (real) candidates.^3^ The number of false positives in the selected critical region can be inferred by comparing the number of target and decoy matches in that region. Different variants of FDR estimation protocols based on concatenated or separate target-decoy search results have been proposed over the years and have become the basis of many postprocessors such as PeptideProphet^4^ or Percolator.^5^

Despite their popularity, decoy-based methods possess some shortcomings. First, they rely on the assumption that it is equally likely for an incorrect match to be mapped to either a decoy or a target candidate,^3^ which strongly depends on the method used to generate the decoy candidates. Second, depending on the specifics of the search algorithm involved, more time may be required to query the experimental data against decoy databases or spectral libraries, especially if the search spaces are large. Finally, they may not perform optimally when there is an insufficient number of data points to model to the right tail of the score distribution of incorrect target matches, which is crucial for accurate and stable FDR estimation (although some strategies have been proposed to mitigate this problem^6^). To address these limitations, several decoy-free approaches have been developed. Typically, such approaches utilize lower-scoring, and therefore incorrect, target matches to enable FDR estimation for the top-scoring target matches (e.g., Nokoi,^7^ expectation-maximization algorithm involving second-best hits,^8^ or modeling lower-order statistics^9^), or derive fixed null models using decoy matches produced for past experimental data (Common Decoy Distributions^10^).

Regardless of the algorithmic details of an FDR estimation method, its accuracy must always be verified or validated. In shotgun proteomics, this process typically involves comparing the FDR estimates with the corresponding values of the actual proportion of false positives in the set of accepted identifications, i.e., the false discovery proportion (FDP).^11^ Validation of FDR estimation methods can be achieved through various approaches utilizing spectra of synthetic peptides,^12^ peptides obtained from a purified protein mix,^13^ or relying on modifying the properties of the query or search spaces involved.^9,14^

One of the most popular validation approaches involves searching spectra against an entrapment database—a collection of known incorrect sequences (entrapments) obtained from the proteome of a foreign organism or via shuffling the sequences of the organism of interest.^15−17^ Since the entrapment database is typically much larger than its counterpart containing potentially correct candidates,^18^ any spectrum that does not have a correct candidate in the search space considered ends up getting matched to an entrapment candidate. In other words, all incorrect spectral matches are ”trapped” by the entrapment database. Those matches can then be used as part of a ground truth data set^17^ or used directly in the assessment of *p*-value calibration.^19^

Entrapment strategies have been primarily used in the context of database searching, where it is relatively straightforward to construct an entrapment database of the desired size and characteristics. Interestingly, this approach has not been as widely adopted in studies involving spectral library searching, perhaps due to the difficulty of constructing an entrapment spectral library that would be large enough while maintaining natural fragment ion patterns in the consensus spectra that are not too similar to those observed for the organism of interest. The issues with validating FDR estimates on spectral library search results led researchers to explore alternative approaches. Recent works^20,21^ have introduced a particularly interesting approach to FDR validation, which deviates from the entrapment-based solutions proposed in database searching scenarios. Instead of appending entrapment spectra (of peptides from foreign organisms) to a spectral library, these studies proposed appending entrapment spectra to the query spectra that are searched against the spectral library. Although this procedure appeared as a simple and effective solution to the problem, our recent work^22^ revealed several of its problematic aspects that ultimately make this approach unsuitable for FDR validation.

One of the crucial issues we uncovered was related to the FDP formula, in which the number of false positives was represented by the number of entrapment matches multiplied by a corrective factor.^20^ This corrective factor, which we called *f*_*g*_, was shown to be consistently too large, which led to an overly conservative assessment of the accuracy of the evaluated FDR estimates. While the idea of using entrapment matches as a proxy for false positives of interest prevents entrapment queries from being used for FDR validation due to inherent conceptual incompatibility, it does not prevent them from being involved in FDR estimation. Therefore, in this work, we propose a new approach for estimating FDR, built upon the existing mix-max procedure,^23^ that incorporates entrapment query matches instead of decoy matches to facilitate the estimation of the number of false positive discoveries within the selected critical region. By leveraging the properties of entrapment queries, our method aims to provide an accurate and reliable estimation of FDR, which could be useful in scenarios where the use of decoys is problematic.

### Methods

#### Estimation of FDR Using the QMM Method

In the formulas presented below, *X*, *Y*, *W*, *Z*, and *Q* are random variables representing the scores of correct (target) matches, highest-scoring incorrect target matches, *max*(*X*, *Y*), highest-scoring decoy matches, and highest-scoring entrapment matches, respectively. The realizations corresponding to *X*, *Y*, *W*, *Z*, and *Q* are denoted as *x*, *y*, *w*, *z*, and *q*, respectively. It is assumed that *Z* ∼ *Y* and *Q* ∼ *Y*, i.e., the scores of decoy matches, entrapment matches, and incorrect sample matches are sampled from the same distribution.

The derivation of the formulas for estimating the number of incorrect matches due to foreign (*F*_0_) and native (*F*_1_) spectra is very similar to the original mix-max method^23^; however, it needs certain modifications to accommodate the use of entrapment queries in lieu of decoys.

The original formula for estimating *F*_0_ is as follows:
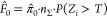
1where π_0_ is the fraction of incorrect matches due to foreign spectra, *n*_Σ_ is the number of all sample spectra (which also equals the total number of decoy matches used in the original mix-max method), and *P*(*Z*_*i*_ > *T*) is the probability of a decoy score *Z*_*i*_ being higher than a selected score threshold *T*, which is the lower bound of the critical region for which *F*_0_ is calculated.

Originally, the above formula was designed to be compatible with the results of a separated target-decoy search using the same set of sample spectra and was implemented in the following form:^23^
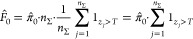
2where 1_*z*_*j*_>*T*_ is an indicator function that equals 1 when the decoy match’s score *z*_*j*_ is higher than the selected threshold *T*; otherwise, it equals 0. In simpler terms, the result of the summation in the equation above represents the number of decoy matches with a score above the threshold *T*.

To enable the use of entrapment queries in the calculation of *P*(*Q*_*i*_ > *T*) replacing *P*(*Z*_*i*_ > *T*), the formula for estimating *F*_0_ must be updated to account for the possibility that the number of entrapment queries may be different from the number of sample spectra considered in the analysis. Therefore, the updated QMM formula for estimating *F*_0_ is as follows:
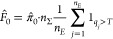
3where *n*_*E*_ is the total number of entrapment queries used, *n*_Σ_ is the number of all sample spectra, and 1_*q*_*j*_>*T*_ is an indicator function that equals 1 when the entrapment query match’s score *q*_*j*_ is higher than the selected threshold *T*; otherwise, it equals 0.

When it comes to estimating the number of incorrect matches mapped to native spectra *F*_1_, its expected value is determined using decoy PSMs in the original mix-max procedure according to the formula:
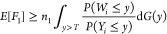
4where *P*(*W*_*i*_ ≤ *y*) is the probability of the observed score *W*_*i*_ of a sample spectrum match being lower or equal to the value *y*, *P*(*Y*_*i*_ ≤ *y*) is the probability of an incorrect score of a sample spectrum match being lower or equal to the value *y*, and *G*(*y*) is the cumulative distribution function of the random variable describing the scores of incorrect matches.

In the QMM method, to make that formula compatible with the use of entrapment query matches, the formula for *E*[*F*_1_] must be updated. As we assumed that *Q* ∼ *Y*, *P*(*Y*_*i*_ ≤ *q*_*j*_) replacing *P*(*Y*_*i*_ ≤ *z*_*j*_) can be estimated as follows:
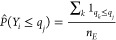
5where *P̂*(*Y*_*i*_ ≤ *q*_*j*_) is the estimate of the probability of a score *Y*_*i*_ of an incorrect sample match being less than or equal to the score *q*_*j*_ of an entrapment match with index *j*, and 1_*q*_*k*_≤*q*_*j*__ is an indicator function that equals 1 when the score *q*_*k*_ of an entrapment match with index *k* is less than or equal to the score *q*_*j*_ of an entrapment match with index *j*, and it equals 0 in all other cases. In simpler terms, the value of *P̂*(*Y*_*i*_ ≤ *q*_*j*_) is determined as the fraction of entrapment matches with scores less than or equal to the score *q*_*j*_ divided by the total number of entrapment matches.

The formula for *P*(*W*_*i*_ ≤ *z*_*j*_) needs to be modified as well. In the original mix-max procedure, it has the following form:
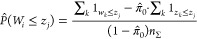
6where 1_*w*_*k*_≤*z*_*j*__ is an indicator function that equals 1 when the score *w*_*k*_ of an observed sample match with index *k* is less than or equal to the score *z*_*j*_ of a decoy match with index *j*, and otherwise equals 0, and 1_*z*_*k*_≤*z*_*j*__ is an indicator function that equals 1 when the score *z*_*k*_ of a decoy match with index *k* is less than or equal to the score *z*_*j*_ of a decoy match with index *j*, and otherwise equals 0.

To make that formula compatible with the use of entrapment queries, the second term in the numerator needs to reflect the estimated number of incorrect matches due to foreign spectra by reference to entrapment query matches instead of decoy matches. It can be achieved by multiplying the second term by *f*_*g*_, i.e., the ratio of sample spectra to entrapment spectra used. Then, the updated formula is as follows:
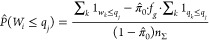
7where 1_*w*_*k*_≤*q*_*j*__ is the indicator function that equals 1 if the score *w*_*k*_ of an observed sample match with index *k* is less than or equal to the score *q*_*j*_ of an entrapment match with index *j*, and it equals 0 in all other cases.

Then, the full formula for the estimated expected value of the number of incorrect matches due to native spectra calculated by using entrapment queries can be represented in the following way:
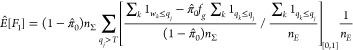
8

The contents of the summation are bound to be in the [0, 1] range to ensure that they maintain the properties of p-values.

After the formulas for estimated *F*_0_ and *F*_1_ and some algebraic operations for simplification were combined, the following formula for the QMM-based FDR estimate can be obtained:
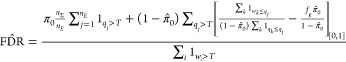
9

In this study, when the QMM method is used to estimate FDR, π_0_ is estimated according to the procedure proposed by Storey and Tibshirani^24^ with the default value of λ = 0.95.

#### Verifying Utility of the Query Mix-Max Method Using Simulated Data Sets

To investigate the validity of the proposed QMM method for FDR estimation, computational experiments involving simulated and real data sets were conducted.

The first part of the investigation is centered on estimating FDR for simulated data sets with different characteristics. Although simulating score distributions of correct and incorrect PSMs is not exactly realistic, it allows us to study scenarios in which it is possible to generate two types of incorrect matches, mapped to foreign and native spectra.^23^ Foreign spectra originate from peptides that are not present in the search space searched; then, any matches made to such spectra are necessarily incorrect. Native spectra, on the other hand, originate from peptides present in the search space used; however, they may also occasionally produce incorrect matches if their correct candidates are randomly outscored by other peptides from the search space. While the extent of occurrence of incorrect matches associated with native spectra seems to have not been studied yet, a simple simulation can be used to show that for extremely well and poorly separated mixture score distributions of correct and incorrect PSMs, the fraction of incorrect PSMs mapped to native spectra is less than 10% and less than 1%, respectively (Supporting Information Note S1). Regardless of which of these percentages is more likely to be observed in the analysis of real data sets and whether they could be considered negligible, it is worthwhile to consider the presence of ”native” incorrect matches in the proposed FDR estimation procedure for the sake of its completeness.

In the construction of simulated data sets with *N* data points, three vectors of scores are sampled from three normal distributions. The first vector, *V*_0_, representing the incorrect matches, has *N* elements *Y*_*i*_ sampled from *N*(0, 1). The second vector, *V*_*E*_, serving as a model of entrapment query scores *E*_*i*_, is sampled from *N*(0, 1) as well and contains  elements, where *f*_*g*_ is the user-specified factor defined as the ratio of sample matches to entrapment matches used in the analysis. The reason why the scores in *V*_0_ and *V*_*E*_ are sampled from the same distribution is that in this simulation study, it is assumed that entrapment queries model the behavior of incorrect sample matches reasonably well. Once the two vectors are generated, *V*_0_ is divided into two sections, with lengths *N*_0_ = ⌊ *N*π_0_ ⌋ and *N*_1_ = *N* – *N*_0_, where *N* is the length of the original vector, and π_0_ is the user-specified fraction of incorrect matches due to foreign spectra. Then, another vector, *V*_1_, corresponding to the scores of correct matches *X*_*i*_, is generated by sampling *N*_1_ elements from the normal distribution *N*(2.5, 1). The first section of *V*_0_ with length *N*_0_ is left unchanged, as it represents the scores of incorrect matches due to foreign spectra. Next, the elements at the same positions in the second section of *V*_0_ with length *N*_1_ and in the *V*_1_ vector are compared against each other, and for each position, max(*Y*_*i*_, *X*_*i*_) is retained. As a result, scores corresponding to correct matches (if *X*_*i*_ ≥ *Y*_*i*_) and ”native” incorrect matches (if *Y*_*i*_ < *X*_*i*_) are generated and form a new vector *V*_*P*_. The final vector of scores, *V*_*F*_, used in the study of FDR estimation comprises the unchanged first section of *V*_0_ with length *N*_0_ and the newly generated vector *V*_*P*_. Then, the QMM procedure is applied to the scores from vector *V*_*F*_ across the 0.1–10% FDR threshold range, and the FDP is calculated based on the ground truth labels known from the simulation process. For each investigated scenario, the process of generating vector *V*_*F*_ and FDR estimation using the QMM method is repeated 100 times, and the mean line along with the 68% pointwise confidence band is recorded.

The simulation study involves investigating three different sets of scenarios. The first set of experiments is based on using different *f*_*g*_ values (between 0.83 and 2), π_0_ = 0.7, and other parameters at default values to investigate how the ratio of sample to entrapment query matches used in the FDR estimation procedure affects the accuracy of the produced FDR estimates. In the second set, the value of π_0_ is changed (between 0.3 and 0.9), while *f*_*g*_ = 2 and other parameters have the default values, as described above. The purpose of these experiments is to evaluate how the fraction of incorrect matches due to foreign spectra affects the accuracy of FDR estimation by the QMM method. Finally, the third set of computational experiments is conducted for various values of the mean of the normal distribution used to generate the scores of correct matches for vector *V*_1_ (mean between 1.6 and 3, while the variance is kept constant at 1), with π_0_ = 0.7, *f*_*g*_ = 2, and other parameters set at default values; the purpose of this investigation is to observe how different degrees of separation between the score distributions of incorrect and correct matches used affect the accuracy of FDR estimates provided by the QMM method.

### Validation by PyViscount

While the analysis using simulated data sets allows for the investigation of clearly defined scenarios under controlled settings, it does not provide any insight into how well the QMM method performs on real data sets that are typically encountered in proteomics practice. To fill this gap, several validation experiments using PyViscount^25^ in a postsearch partition mode were conducted on the QMM method. Three data sets of real experimental spectra were used: the *S. cerevisiae* (SC) data set (PXD014189, Orbitrap Fusion Lumos, 109,880 MS2 spectra), the *Cricetulus griseus*, i.e., Chinese hamster (CH) data set (PXD008760, Q Exactive HF Orbitrap, 20,683 MS2 spectra), and the *E. coli* (EC) data set (PXD002912, Orbitrap Velos, 43,063 MS2 spectra). We selected all data sets acquired on Orbitrap instruments to make sure that the entrapment and sample data sets have similar basic characteristics and to reduce the confounding effects due to the different types of mass spectrometers used to obtain the experimental spectra. These data sets were searched using the Tide search engine (part of Crux^26^) with default settings, against the respective protein sequence databases downloaded from UniProt (UP000002311 for SC, UP000001075 for CH, and UP000000625 for EC data sets) and processed using tide-index.^27^ Each of these data sets had its FDR estimated across the FDR threshold range of 0.1–10% using the QMM method executed on two different entrapment data sets: *Pyrococcus furiosus* (PT) (PXD001077, Orbitrap Velos, 15,023 MS2 spectra) and *Arabidopsis thaliana* (AT) data set (PXD001207, Orbitrap Velos, 27,840 MS2 spectra), also searched by Tide with default settings against the protein sequence database corresponding to the analyzed sample data set’s organism. The PyViscount validation results (proxy FDP vs FDR) were provided for FDR estimated at the PSM and peptide levels in the form of optimal single-line plots, supplemented with the respective bootstrapped 68% confidence bands.

To place the results of this analysis in a proper context, the FDR estimation accuracy of the QMM method was benchmarked against analogous results obtained for FDR estimated using target-decoy competition (decoy method)^3^ and PyLord (decoy-free method).^9^ To obtain TDC-based estimates, the selected data sets were first searched against the corresponding concatenated target-decoy protein sequence databases using Tide with default settings; the decoy component of each database was generated using the “tide-index” tool from the Crux suite with default settings. Then, the FDR was estimated according to the formula  where *D* and *T* are the numbers of decoy and target matches found in the selected critical region, respectively.^28^

To obtain PyLord-derived FDR estimates, the results of searching the analyzed data sets against the respective target protein sequence databases were provided as input to PyLord and processed with default settings. The parameters of the top null models returned by PyLord were then used as input to PyViscount and used to calculate p-values for all PSMs, which were then employed in FDR estimation using Storey’s method.^29^

## Results

### Simulation Study

**Figure 1:**
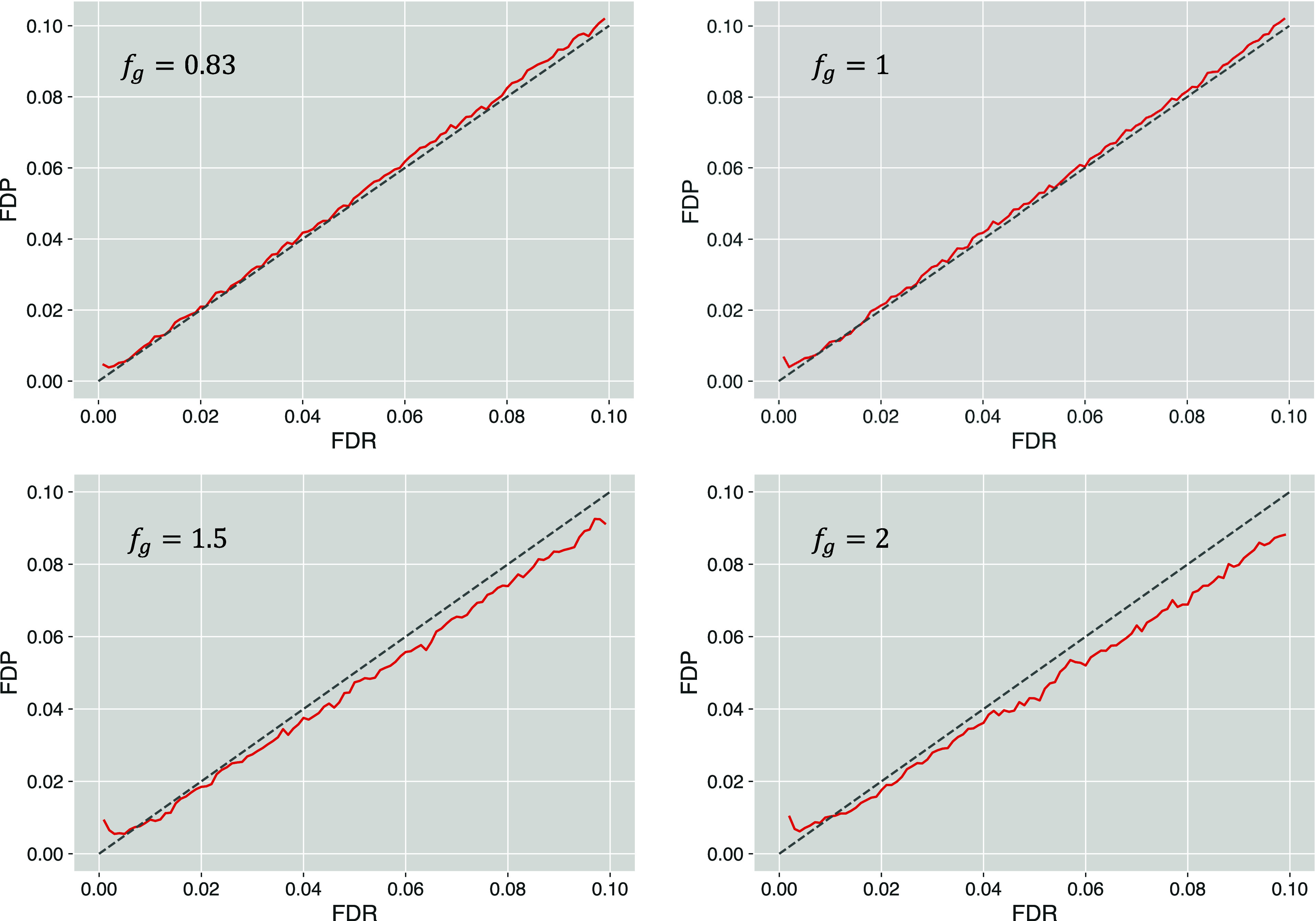
FDP vs FDR results obtained using the QMM method for the simulated sample and entrapment data sets associated with different *f*_*g*_ values.

The study of FDR estimates produced in scenarios with different *f*_*g*_ values reveals that a smaller ratio of sample to entrapment spectra used in the procedure favors more accurate FDR estimation (Figure 1). In the cases with *f*_*g*_ = 0.83 and *f*_*g*_ = 1, the estimated FDR is very close to the actual FDP values, although a minimally liberal tendency can be observed in those cases. Some deviation from the ideal values is expected since the π_0_ used in the QMM formula is not exact but is estimated as well, contributing to the overall variability of the QMM-based FDR estimates. As *f*_*g*_ is increased to 1.5, the QMM method tends to generally become more conservative, which is more evident in the range of FDR values above 3%. A similar FDR overestimation trend can be observed even more clearly for the scenario with *f*_*g*_ = 2. At the same time, a slight tendency to underestimate FDR is revealed at very low FDR values (<1%). This kind of liberal bias appears mainly due to the scarcity of entrapment data points in the overlap region between the score distributions of correct and incorrect matches and becomes more pronounced when larger *f*_*g*_ values are used. Therefore, to avoid liberal FDR bias, it is recommended that *f*_*g*_ ≤ 1 be kept in real-life applications of the QMM method. From a practical perspective, the slight liberal bias at very low FDR values does not seem to be too problematic, because around the commonly used 1% FDR value, the estimation is quite accurate, and at larger FDR values, the moderate conservative estimation trend will slightly reduce the number of true positives accepted for further analysis, while still maintaining stringent FDR control. Therefore, these results suggest that the QMM method could provide reasonably accurate FDR estimates in various *f*_*g*_ scenarios, with a safely conservative tendency in more challenging cases when the number of entrapment queries available is scarce.

**Figure 2:**
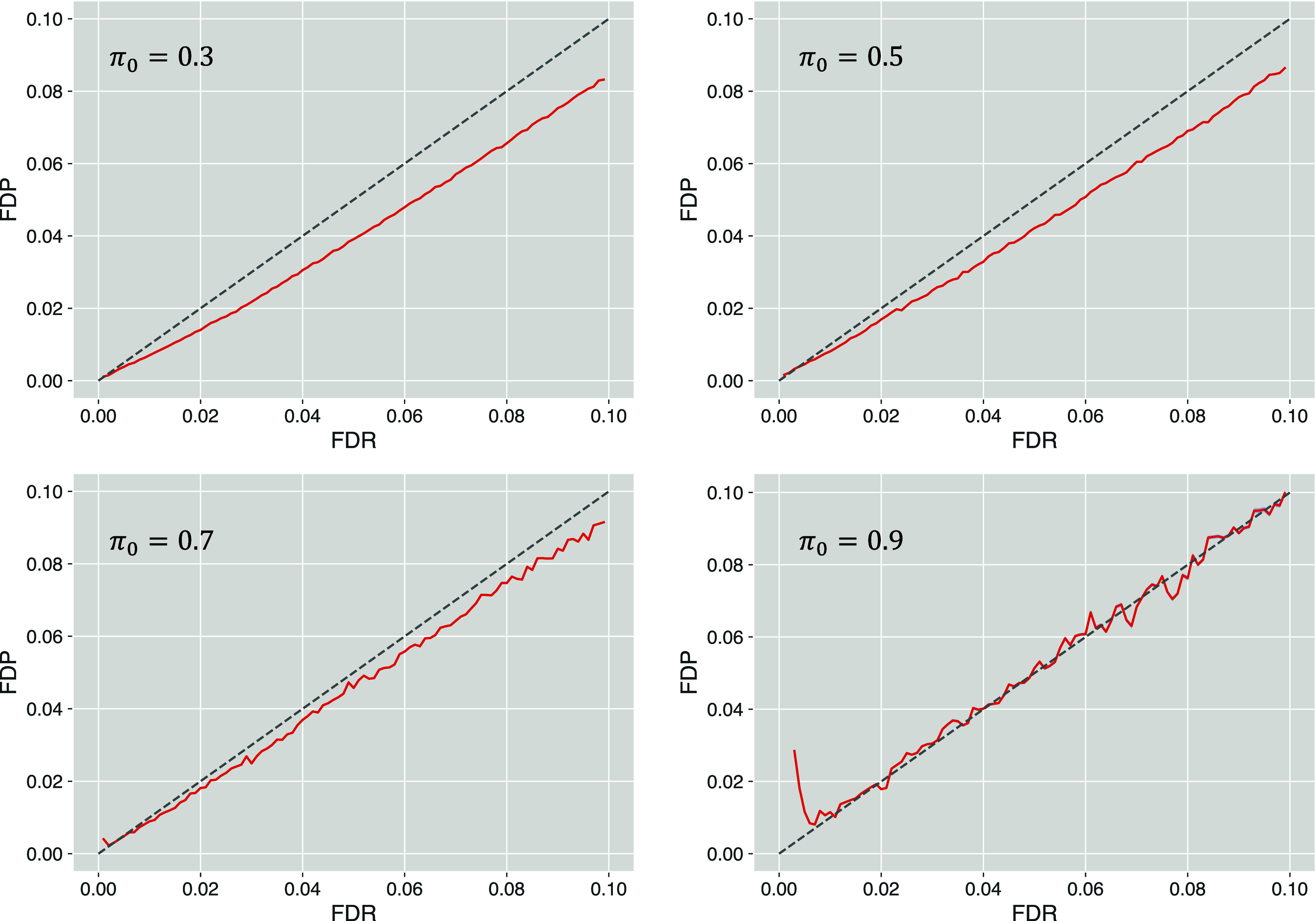
FDP vs FDR results obtained using the QMM method for the simulated sample and entrapment data sets associated with different π_0_ values.

Results of FDR estimation by the QMM method in scenarios with different π_0_ values (examples of histograms shown in Supplementary Figure S1) indicate that a higher fraction of incorrect matches due to foreign spectra present in the analyzed data set is associated with more accurate FDR estimates (Figure 2). At π_0_ = 0.3 and 0.5, the QMM provides conservative FDR estimates across the whole evaluated range of FDR values. The tendency to overestimate the FDR is noticeably reduced for the scenario with π_0_ = 0.7. When the value of π_0_ equals 0.9, the FDR estimates produced by the QMM method become very accurate and remain close to the FDP counterparts for most of the evaluated FDR values, although they tend to be associated with more variability than the values from scenarios with smaller π_0_. To some extent, the increased variability could be explained by the fact that data sets characterized by large π_0_ have relatively few correct PSMs considered in the calculation of FDR. As a result, particularly in the lower FDR region, even a small difference in the number of entrapment matches falling in the evaluated critical region may have a substantial impact on how much the final FDR estimate can vary. Another factor that could contribute to the slightly inaccurate FDR estimates is that the QMM method includes not only the estimated number of false positives inferred from the number of entrapment queries but also the estimated π_0_ value that may be associated with some estimation error. Nevertheless, the results of FDR estimation for scenarios with different π_0_ values seem to suggest that the proposed QMM method could be a viable FDR estimation alternative, and the modifications introduced to the original mix-max procedure do not render the updated version useless.

**Figure 3:**
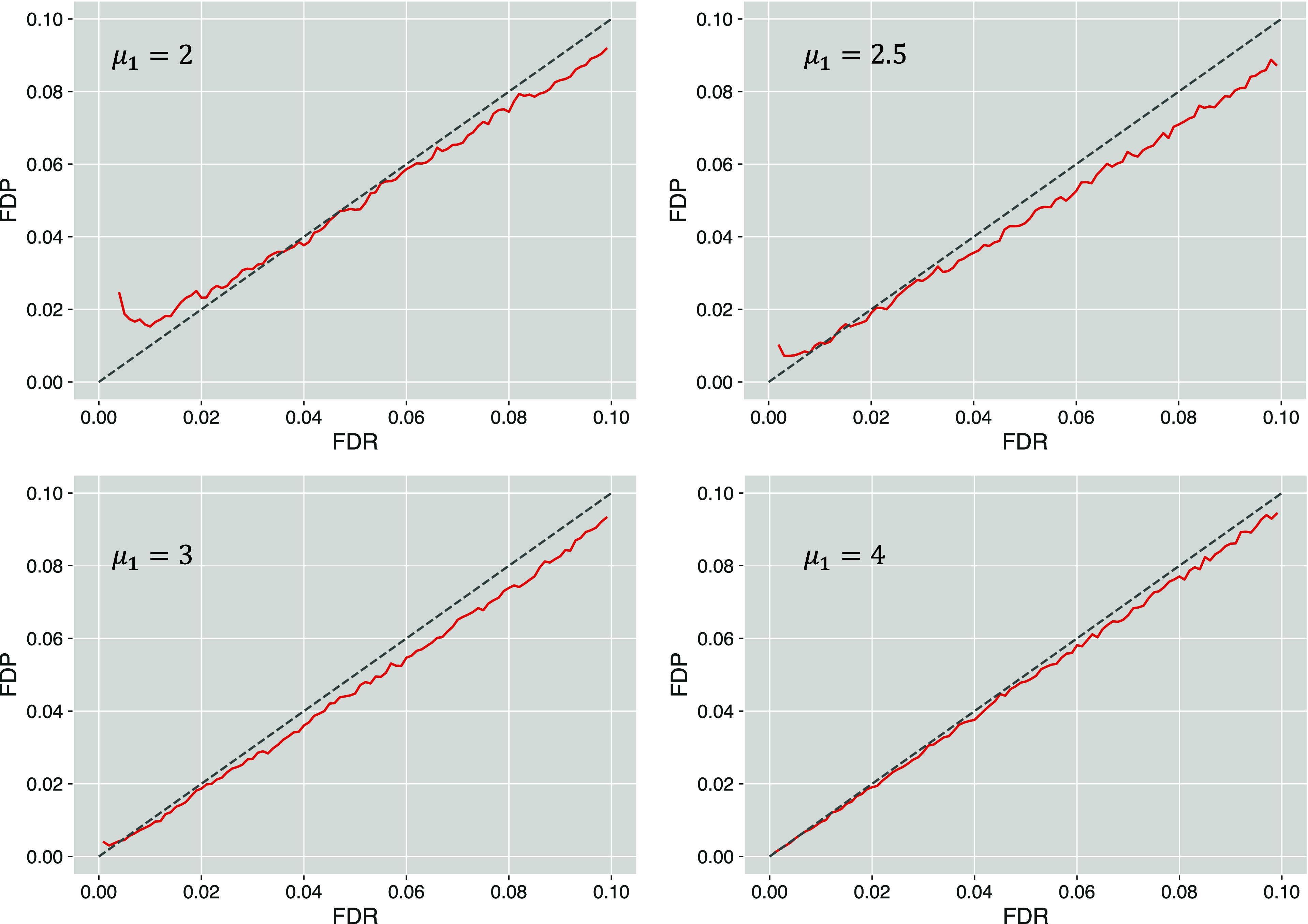
FDP vs FDR results obtained using the QMM method for simulated sample and entrapment data sets associated with different values of the mean (μ_1_) of the score distribution of correct simulated sample matches.

The computational experiments conducted on score distributions with different degrees of separation between scores of correct and incorrect matches reveal that a larger distance between the two components of the mixture distribution leads to more accurate FDR estimation (Figure 3). For the scenario with μ_1_ = 2, the QMM method underestimates FDR for the lower FDR threshold (0.1–2.5% threshold range) and tends to slightly overestimate FDR for the higher FDR thresholds (above 6% threshold). The underestimation trend seems to be reduced for μ = 2.5, while the overestimation is also displayed at lower FDR thresholds (above the 3% threshold). The tendency to provide overly liberal FDR estimates at the lower threshold values when the score distributions of correct and incorrect matches are poorly separated could be explained by the fact that the ”actual” *f*_*g*_ at the sparse right-tail region of the score distribution of incorrect sample matches (called the incremental factor *f*_*in*_ in our previous work^22^) may fluctuate substantially, often below the value of the global *f*_*g*_ factor. In such cases, the utilization of the global *f*_*g*_ factor in the QMM formula may result in an excessively large number of estimated false positives at the evaluated critical region. However, once the separation between the correct and incorrect matches is increased, the number of correct and incorrect matches used in the formula for FDR is increased, producing more accurate estimates of probability values used in the QMM formula and leading to more accurate FDR estimates even at the lower range of FDR thresholds. The trend of improved accuracy of the FDR estimation can be observed for μ_1_ = 3 and very clearly for μ_1_ = 4. Considering the fact that the separation associated with μ_1_ = 2 or μ_1_ = 2.5 is extremely poor (Supplementary Figure S2), and the data generated nowadays with newer mass spectrometers and improved identification algorithms are closer to the scenario with μ_1_ = 4, the QMM protocol, despite its limitations in more challenging separation scenarios, could be practically useful in FDR estimation of more common score mixture distributions with decently separated components of correct and incorrect matches.

### Validation by PyViscount

**Figure 4:**
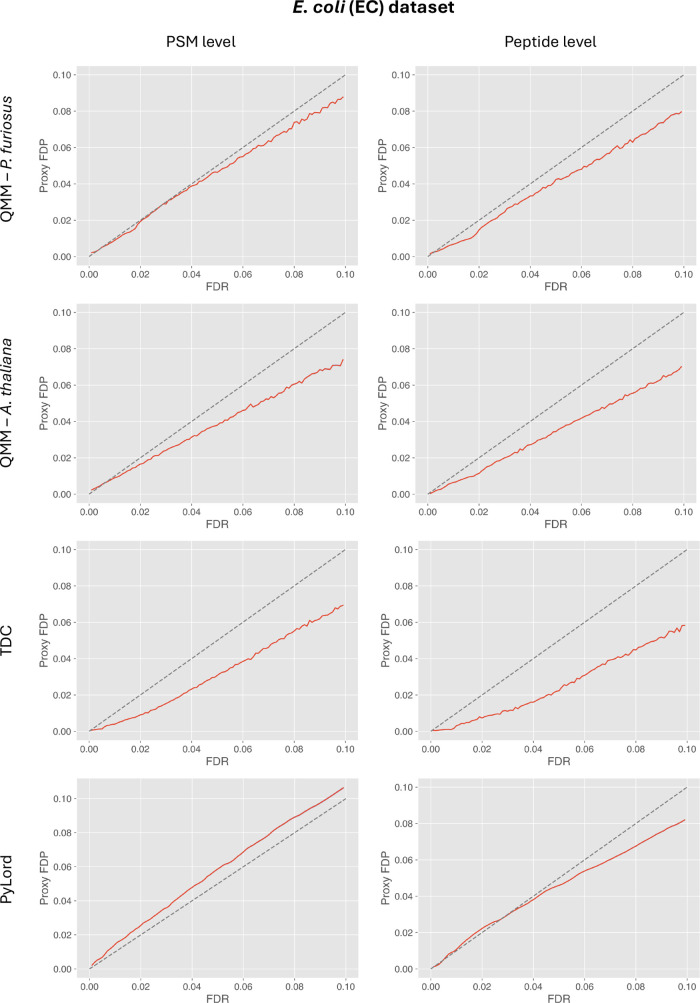
Results of validating FDR estimation at PSM (left column) and peptide (right column) levels using the QMM method, TDC, and PyLord on the *E. coli* sample data set. Entrapment queries required by the QMM method were taken from the *P. furiosus* (top row) and *A. thaliana* data sets. Each plot exhibits the comparison of PyViscount-determined proxy FDP vs FDR estimated by the evaluated methods along with the *x* = *y* line (dashed).

The estimation of FDR using the QMM method with the PF data set is quite accurate, with a conservative tendency displayed for larger FDR values (Figure 4). At the PSM level, the estimated FDR is very close to the actual FDP values in the range of 0.1–4% FDR thresholds, with slight overestimation above 4% FDR. At the peptide level, there is a clear conservative trend observed throughout the whole evaluated FDR range, but the magnitude of that effect seems to increase with an increase in the estimated FDR. This is not necessarily an undesirable trend because it suggests that (at least in this particular setting), the entrapment query-based FDR estimation can ensure FDR control at the user-specified level. When the AT data set is used to provide entrapment queries for the analysis using the QMM method, similarly conservative FDR estimates are obtained (Figure 4). However, the FDR estimation trend is stronger (with a larger downward bias) at both the PSM and peptide levels, when compared to the analogous data obtained for the PT data set.

A strong conservative FDR estimation tendency is also observed for the results obtained using the popular target-decoy competition method (Figure 4). The TDC-based estimates are associated with substantial downward bias that can reach around 3 and 4 percentage points for FDR = 10% at the PSM and peptide levels, respectively. Since both the QMM and TDC methods share some similarities (e.g., using decoy or entrapment query “counting” for estimating the number of false positives), it is not surprising that they return similar FDR estimates. This also indicates that the QMM method could be considered a viable alternative to TDC since it provides slightly more accurate FDR estimates while still allowing for consistent FDR control. In fact, the results provided by the QMM method are more accurate than those of the decoy-free estimation by PyLord, which tends to slightly underestimate the FDR at the PSM level but provides more accurate FDR estimates at the peptide level.

**Figure 5:**
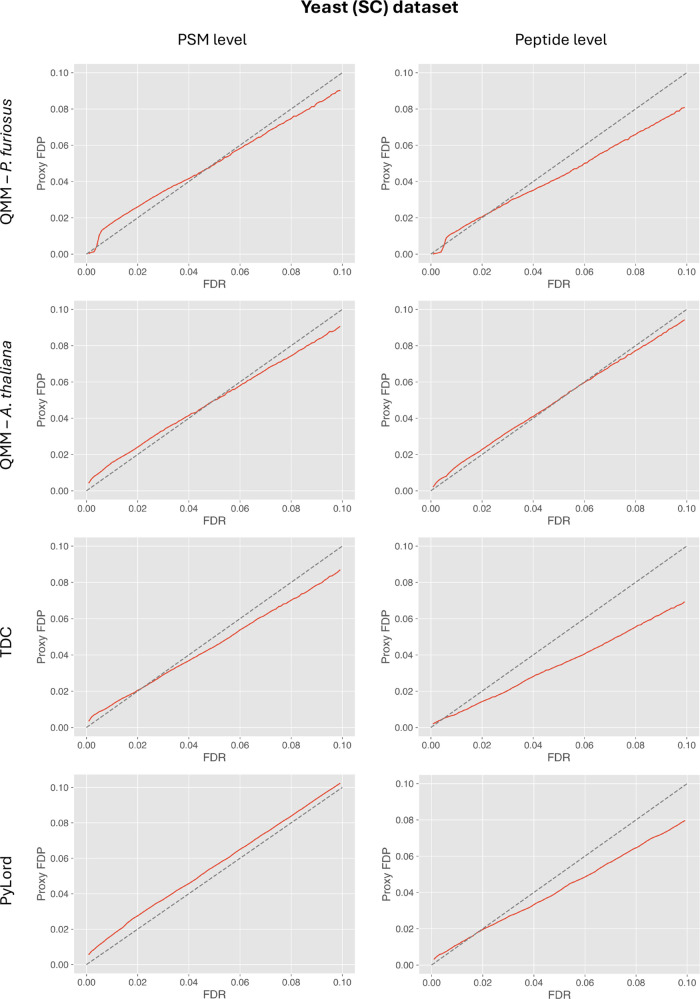
Results of validating FDR estimation at PSM (left column) and peptide (right column) levels using the QMM method, TDC, and PyLord on the *S. cerevisiae* sample data set. Entrapment queries required by the QMM method were taken from the *P. furiosus* (top row) and *A. thaliana* data sets. Each plot exhibits the comparison of PyViscount-determined proxy FDP vs FDR estimated by the evaluated methods along with the *x* = *y* line (dashed).

In the analysis of the SC data set, at the PSM level, the FDR is underestimated at lower (below 5%) and slightly overestimated at higher FDR values (above 5%) when the PT data set is used to provide entrapment queries to the QMM method (Figure 5). At the peptide level, there is a minimal tendency to provide liberal FDR estimates below the 3% threshold and more conservative estimates above that threshold, with the downward bias reaching 2% for FDR = 10%.

A more accurate FDR estimation is demonstrated by the QMM method when the AT data set is used (Figure 5). At the PSM level, the variant with the AT data set exhibits an almost identical estimation trend as the already discussed PT variant (slight underestimation below the 5% and overestimation above the 5% FDR threshold). However, at the peptide level, the QMM method with the AT data set provides more accurate FDR estimates across the whole FDR range evaluated.

The fact that the QMM method provides similar FDR estimation at the PSM level but not at the peptide level when the PT and the AT data sets are used could be caused by the difference in the complexity of the corresponding proteomes. The *P. furiosus* proteome consists of 2044 proteins, while the *A. thaliana* proteome has 39,279 proteins. As a result, when both entrapment query data sets are used at the PSM level, there are sufficiently many data points to estimate FDR in both cases, so the differences between FDR estimates in these scenarios are not particularly large. However, at the peptide level, only one top-scoring PSM per peptide is considered, which may favor a more accurate estimation performance when the AT data set is used.

At the PSM level, the TDC-based FDR estimates are, similarly to the QMM variants, slightly underestimated below the 3% FDR threshold and tend to be more conservative above that threshold (Figure 5). At the peptide level, however, the underestimation tendency becomes much stronger, resembling what was observed for the EC data set.

The similarity between the results obtained for this yeast data set and the already discussed EC data set is also evident when PyLord was used to estimate FDR. At the PSM level, the PyLord estimates were consistently slightly liberal, while the peptide-level ones got increasingly conservative above the 2% FDR threshold (Figure 5).

When the TDC- and PyLord-derived FDR estimation results are compared with those obtained using the QMM method with the PT and the AT data sets, it is evident that the QMM variants often provide more accurate FDR estimates. In fact, the QMM variant supplemented with the AT data set exhibited the most consistent and accurate FDR estimation at both PSM and peptide levels among all the evaluated methods. Therefore, based on the results obtained for the analysis involving the SC data set, it seems reasonable to consider the QMM method as a viable alternative to other existing decoy-based and decoy-free FDR estimation protocols in shotgun proteomics.

**Figure 6:**
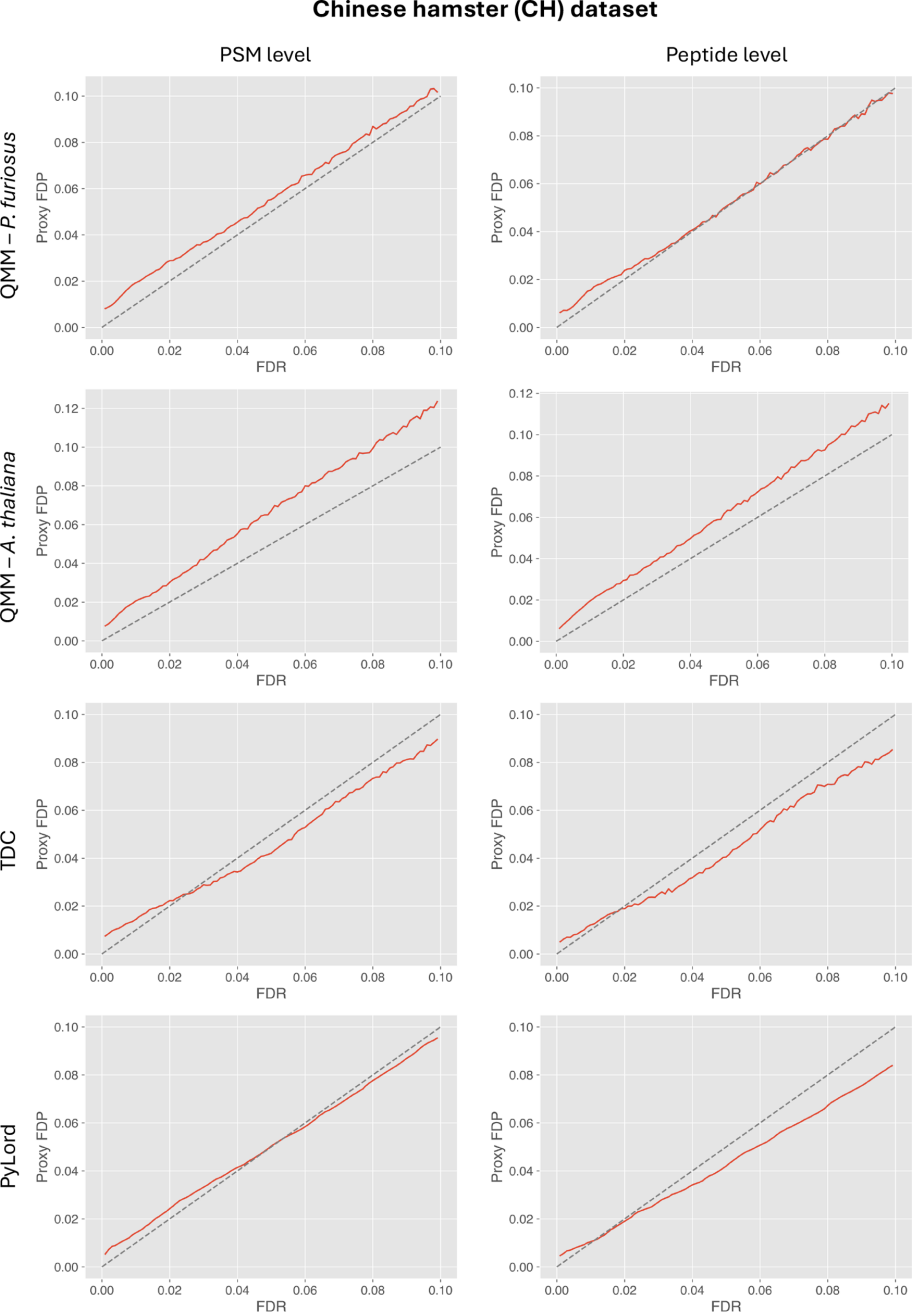
Results of validating FDR estimation at PSM (left column) and peptide (right column) levels using the QMM method, TDC, and PyLord on the *C. griseus* sample data set. Entrapment queries required by the QMM method were taken from the *P. furiosus* (top row) and *A. thaliana* data sets. Each plot exhibits the comparison of PyViscount-determined proxy FDP vs FDR estimated by the evaluated methods along with the *x* = *y* line (dashed).

However, the QMM method is not always superior or on par with the existing decoy and decoy-free methods. As shown in the results of the analysis conducted on the CH data set, the QMM method using the PT data set exhibits a consistent FDR underestimation trend at the PMS level, with the upward bias decreasing for higher FDR values (Figure 6). While a minimally liberal tendency is also displayed for the peptide-level analysis, above the 3% FDR threshold, the estimates become very close to the actual FDP values.

An underestimation trend at the PSM level is also exhibited by the QMM method when the entrapment queries are taken from the AT data set (Figure 6). However, in this case, the upward bias seems to increase for larger FDR values. A very similar trend is observed for the peptide-level analysis as well. These results obtained for both variants of QMM demonstrate that at least in some scenarios, the FDR estimates produced in different settings are strongly influenced by the nature of the entrapment query data set used in the procedure. This observation is consistent with the results of the analysis presented in our previous paper, where both AT and PT data sets differ in terms of their p-value distributions or the trends in the incremental factor *f*_*in*_ values (which in turn provide information about the accuracy of the *f*_*g*_ factor used).^22^

In terms of comparison with the existing alternatives, i.e., TDC and PyLord, it is clear that those alternatives generally provide more accurate (and sometimes a bit more conservative but preferable to the overly liberal) FDR estimates than the QMM variants. At the PSM level, both TDC and PyLord tend to slightly underestimate FDR for lower values (below 2 and 5%, respectively); however, at the higher values, they effectively control FDR at the desired threshold, with PyLord being the more accurate solution (Figure 6). A similar trend is exhibited at the peptide level, although in this case, PyLord appears more conservative above 2%, while both methods are fairly accurate below the 2% FDR threshold.

Considering all the analysis results generated for the CH data set, it is evident that the QMM method requires careful examination of the nature of the data set subjected to the FDR estimation procedure and the data sets from which the entrapment queries are taken. The PF data set may provide more accurate FDR estimates in most of the evaluated scenarios because *P. furiosus*—an *Archaea* species*—*is more evolutionarily distant from the organisms of origin for the data sets used in this study than *A. thaliana—*a eukaryote. As a result, the peptides represented in *P. furiosus* data sets are less likely to be highly homologous or shared with the peptides of the analyzed organism and thus less likely to violate the assumptions underpinning the QMM method. This intuition appears to be supported by a past study, which recommends *P. furiosus* as a useful reference organism to be used in the analysis of proteomics data.^30^

## Conclusions

In this work, the relatively recent entrapment-question-based mode of analysis, originally used for validating FDR estimates, was repurposed to become the foundation of an alternative FDR estimation method. The newly proposed protocol, called the query mix-max (QMM) method, is a modified version of the existing mix-max procedure designed for FDR estimation using target-decoy search results. The QMM method utilizes entrapment query matches instead of decoy matches to facilitate the estimation of the number of false positive discoveries in the evaluated critical regions. A series of computational experiments conducted on simulated and real data sets demonstrates that the QMM method is capable of providing reasonably accurate FDR estimates across various scenarios and could be considered a viable alternative to other existing FDR estimation protocols such as target-decoy competition or PyLord.

While the computational experiments presented in this work confirm the promising FDR estimation performance of the QMM method, the proposed procedure still has some limitations. Since the QMM protocol involves counting individual instances of entrapment query matches to enable estimation of the number of false positive sample matches, it requires the user to provide a reasonably large number of entrapment queries to allow reasonably stable FDR estimates, especially for low FDR values. The reason behind this requirement is that the right tail of the score distribution of incorrect PSMs, crucial for the FDR estimation, is typically sparse; thus, the fewer data points are available to model that tail, the less precise the FDR estimates provided by counting the PSMs in the critical region become. Another limitation of the QMM method is the narrowed-down scope of application, which has not been adapted to accommodate the possibility of using search results from open-search scenarios, where peptide candidates can be associated with various modifications. Similarly, the occurrence of chimeric spectra and the problem of their inclusion in the estimation of FDR have not been considered in the development of the QMM framework yet. Another limitation of the proposed framework is that the extent to which the mass spectrometers used to acquire the entrapment and sample data sets need to be similar is unknown. Furthermore, the QMM method requires the users to rely on their expertise to select an optimal entrapment organism for the analysis of a data set coming from a particular sample organism. Even though the data set obtained for *P. furiosus* generally seems to work well in most of the investigated scenarios, it does not necessarily mean it will provide optimal performance for any kind of sample organism. Lastly, while the QMM method is built upon the existing mix-max framework, it employs entrapment query matches instead of the well-researched decoy matches, and due to how these matches are generated, any theoretical guarantees related to the accuracy and bias of FDR estimation by QMM remain to be explored.

The discussed limitations naturally lead to the recognition of the need for further research to be conducted on the utility of entrapment query-based FDR estimation, in the form of the QMM method and possibly beyond it. In terms of the problem of selecting the most suitable entrapment organism for analysis, it would be beneficial to conduct a deeper investigation into how the evolutionary distance between the sample and entrapment organisms impacts the FDR estimation process. In particular, it would be desirable to work out a more standardized procedure for selecting an optimal entrapment data set according to straightforward guidelines that could be followed by researchers with minimal experience in proteomics data analysis. Furthermore, a study of more extreme scenarios might provide useful insights into the range of conditions under which the QMM method remains a reliable choice for accurate FDR estimation. For instance, the question of what the minimal number of entrapment queries (or the minimal value of *f*_*g*_) is that could still provide accurate FDR estimates could be worth investigating, with possible connections made to the already researched proposal of using a target-small decoy search strategy in the realm of decoy-based FDR estimation methods.^31^ Finally, the properties and potential modifications of the QMM method to make it compatible with open-search results and chimeric spectral matches could make the FDR estimation method more versatile and attractive to proteomics practitioners.

## Supporting Information Available

Supporting simulations; simulated fraction of incorrect matches due to native spectra; examples of simulated mixture score distributions for different π_0_ values; and examples of simulated mixture score distributions for different μ_1_ values (PDF)
