# Supplementary material for: Query Mix-Max Method for FDR Estimation Supported by Entrapment Queries
Source: J Proteome Res. 2025 Feb 5;24(3):1135–47. doi: 10.1021/acs.jproteome.4c00744 (PMC11894652; doi:10.1021/acs.jproteome.4c00744)
Supplement: Supplementary file 1 — pr4c00744_si_001.pdf [file pr4c00744_si_001.pdf]

Supporting Information for the article: ”Query Mix-Max Method for FDR Estimation Supported by Entrapment Queries”

Dominik Madej, Henry Lam

Department of Chemical and Biological Engineering, the Hong Kong University of Science and Technology, Hong Kong, China

Content

Supplementary Note S1: Supporting simulations

Supplementary Table S1: Simulated fraction of incorrect matches due to native spectra

Supplementary Figure S1: Examples of simulated mixture score distributions for different π_0 values

Supplementary Figure S2: Examples of simulated mixture score distributions for different μ_1 values

Supplementary Note S1 Supporting data for the entrapment query study

The proposed query mix-max method takes into account incorrect matches due to foreign and native spectra. According to the nomenclature outlined in [2], the foreign spectra represent peptides that are not present in the selected search space due to a variety of reasons, e.g., the spectra may represent non-peptide species, contaminants, or contain modifications that are unaccounted for. Therefore, any matches made to the foreign spectra turn out to be incorrect, and they are typically described by a parameter quantifying the fraction of such matches in the whole analyzed dataset called π_0, PIT, or p_0 [1]. Native spectra, on the other hand, represent peptides that are presented in the search space considered. However, sometimes an incorrect peptide candidate may outscore the correct one in the matching process and a native spectrum gets assigned with a wrong peptide giving rise to an incorrect match. While investigating this phenomenon is difficult to conduct on real experimental datasets, it can be analyzed in-depth using simulated data.

To provide a rough idea of what fraction of incorrect matches could be attributed to native spectra, the following computational experiment was conducted. First, a set of 10,000 scores was sampled from a normal distribution $N(0,1)$ and placed in a vector V_0. Then, another set of 10,000 scores was sampled from one of the following normal distributions $N(\mu_1,\sigma_1)$ (with μ_1 between 2 and 4 and σ_1 equal to 1) and placed in a vector V_1. Each of these normal distributions corresponded to a different analysis scenario. The value of the π_0 parameter was between 0.3 and 0.9 to represent scenarios with different fractions of incorrect "foreign" matches. To construct vector V_F with the final scores, the following procedure was executed. The first section of vector V_0 of length $N_0 = \lfloor N * \pi_0 \rfloor$ was appended to V_F in an unchanged form. The elements in the second part of vector V_0 were compared to elements with the same positions in vector V_1. For each position, the larger score was

selected and appended to V_F. The label of each score in V_F was determined based on its origin. If the score was taken from the first portion of V_0 with length N_0, it was marked as an incorrect foreign match. If the score originated from the second section of V_0, it was marked as an incorrect native match. The remaining scores (that were taken from V_1) were considered correct matches. In each investigated scenario, the fraction of incorrect matches due to native spectra among all incorrect matches D was calculated.

There were two sets of simulations executed. The first set involved $\pi_0 = 0.7$ and different values of μ_1: 2, 2.5, 3, and 4. All the other parameters were used with their default values. The purpose of this part of the investigation was to obtain the D values in scenarios with different degrees of separation between score distributions of incorrect and correct matches. The second set of simulations had $\mu_1 = 3$ and different values of π_0: 0.3, 0.5, 0.75, and 0.9. This study was designed to obtain the D values in scenarios where different fractions of incorrect matches due to foreign spectra were present. Each of the simulated cases was repeated 200 times, and the average and standard deviation values of D were calculated.

As shown in Supplementary Table S1, The largest value of D was obtained for the scenario with $\mu_1 = 2$ and $\pi_0 = 0.7$ and it was equal to 7.88%. It is not a particularly high value considering the extremely poor separation between the score distributions of correct and incorrect matches as visualized in Supplementary Figure S2. As the value of μ_1 increased, D got steadily smaller, reaching only 0.232% for the scenario with $\mu_1 = 4$, which is more representative of the score distributions obtained by the latest search engines and post-processors. The decrease in D with an increase in μ_1 is expected because a larger separation between correct and incorrect score distributions implies that the chance of an incorrect candidate outscoring the correct one for a native spectrum becomes much smaller. The values of D for the simulated scenarios with different π_0 (as visualized in Supplementary Figure S1) are minimally different from each other, which suggests that parameter π_0 has negligible impact on the fraction of incorrect matches due to native spectra

μ_1	π_0	$\hat{D}$	standard deviation of D
2	0.7	0.0788	0.00488
2.5	0.7	0.0383	0.00380
3	0.7	0.0168	0.00238
4	0.7	0.00232	0.000853
3	0.3	0.0172	0.00165
3	0.5	0.0170	0.00200
3	0.75	0.017316	0.00263
3	0.9	0.0168	0.00399

Supplementary Table S1: The values of the fraction of incorrect matches due to native spectra D for simulated scenarios with different mean of the distribution of correct matches (μ_1) and the fraction of incorrect matches due to foreign spectra (π_0).

that arise in the simulated datasets.

While the simulated datasets may not fully capture the characteristics of correct and incorrect matches that are produced for real experimental datasets, it seems reasonable to assume that the D values obtained for such datasets are not drastically different from those calculated on the basis of simulated results. In the context of FDR estimation, it means that accounting for the presence of incorrect matches due to native spectra is a nice addition making the estimation more accurate and comprehensive, but the magnitude of that improvement may not be very noticeable in practice.

Supplementary Figure S1: Examples of simulated mixture score distributions for different π_0 values.

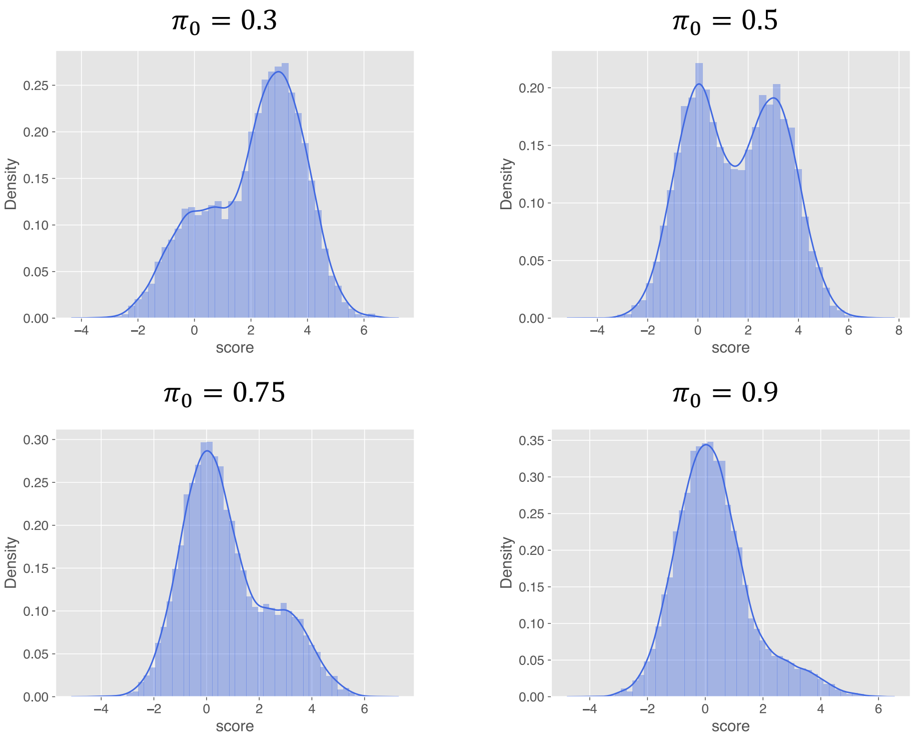

Supplementary Figure S2: Examples of simulated mixture score distributions generated based on different starting distributions.

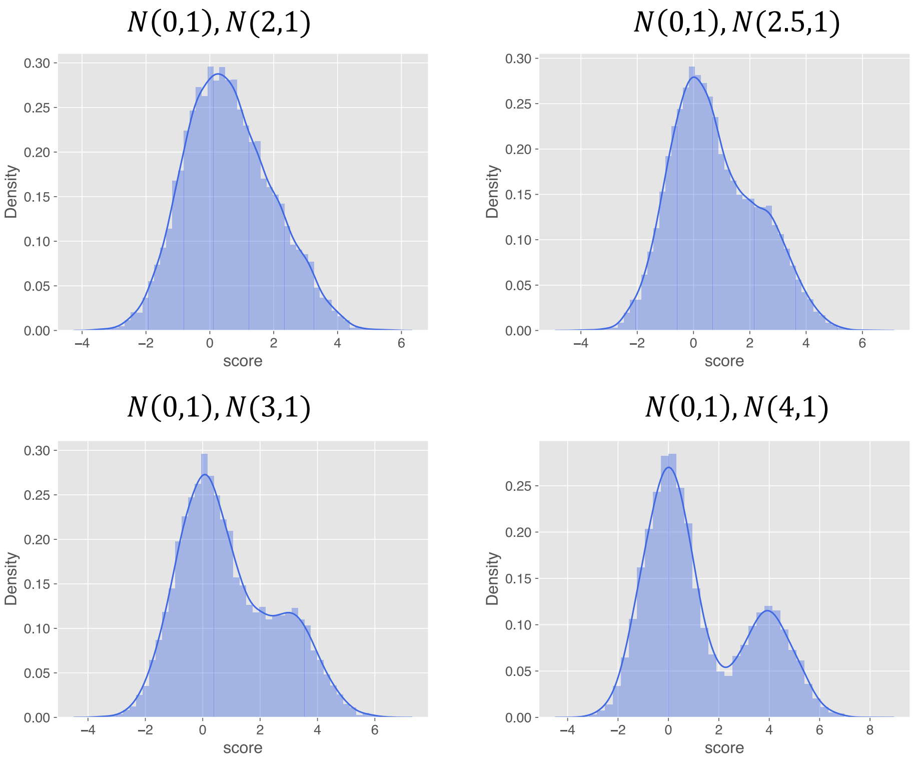

References

[1] Lukas Käll, John D Storey, Michael J MacCoss, and William Stafford Noble. Assigning significance to peptides identified by tandem mass spectrometry using decoy databases. *Journal of proteome research*, 7(01):29–34, 2008.

[2] Uri Keich, Attila Kertesz-Farkas, and William Stafford Noble. Improved false discovery rate estimation procedure for shotgun proteomics. *Journal of proteome research*, 14(8):3148–3161, 2015.
